# Supplementary material for: Cost-effectiveness of leveraging existing HIV primary health systems and community health workers for hypertension screening and treatment in Africa: An individual-based modeling study
Source: PLoS Med. 2025 Jan 24;22(1):e1004531. doi: 10.1371/journal.pmed.1004531 (PMC11805449; doi:10.1371/journal.pmed.1004531)
Supplement: S1 Text — In this file, we sample from the 3000 model runs based on charactersitics at study baseline (end 2023) that are similar to national and subnational settings in Uganda. We provide estimates of health effects, cost, and cost-effectiveness, scaled to the population of Uganda. The purpose of this sample country profile is to illustrate how model findings can be used to identify expected outcomes for a particular setting. (PDF) [file pmed.1004531.s002.pdf]

Country Profile: Uganda

Estimated Effect of Hypertension Treatment Policies on Hypertension treatment and cardiovascular disease in Uganda from 2024 to 2074

Methods

We scaled model output to a population size of 26,029,383 persons ≥15 years of age in 2022 (World Bank).[1] We selected from a total of 3000 setting-scenarios 1,398 [47%] which matched regional and national demograhic and health data from Uganda. Criteria for selection were model setting-scenarios with:

- Hypertension prevalence <32% among adults ≥18 years
- Hypertension diagnosis <26% among adults ≥18 years
- HIV prevalence <19% among persons aged 15-49 years

Population size estimate

Based on model estimates for Uganda

Mean (90% range)	2023	2073
Adults aged 18+	23.6 million (23.2 – 23.9 million)	48.4 million (37.1 – 62.4 million)
Adults aged 40+	9.1 million (7.3 – 11.0 million)	25.9 million (21.2 – 31.7 million)

Current state of hypertension treatment

	**2023 (model)** Mean (90% range)	**Literature (Guwatudde 2015; Geldsetzer 2019)**[2,3]
Prevalence	25% (19 to 31%)	26.5% (23.3 to 28.5%)
Diagnosis	19% (14 to 25%)	18.1% (15.2 to 21.2%)
Treatment	3% (2 to 5%)	7.5% (5.8 to 9.5%)
Control	2% (1 to 3%)	2.3% (1.5 to 3.4%)

HIV prevalence

	**2023 (model)** Mean (90% range)	**Uganda AIDS Commission 2021**[4]
HIV prevalence among adults 15-49 years	7% (1 to 17%)	National: 5.4%<br>District prevalence: 0.2% (Nabilatuk) to 18.8% (Kalangala)

Hypertension treatment over next 50 years under three policies

	Age	Continuation of current policies	Primary care hypertension treatment	Population-level CHW screening
Prevalence (SBP ≥140)	18+	28% (20% to 35%)	28% (20% to 35%)	28% (20% to 36%)
	45-64	42% (28% to 53%)	42% (28% to 53%)	42% (27% to 53%)
Diagnosis	18+	20% (15% to 25%)	23% (17% to 29%)	56% (48% to 62%)
	45-64	24% (18% to 31%)	28% (20% to 36%)	78% (71% to 85%)
Current treatment	18+	4% (2% to 6%)	12% (7% to 17%)	37% (30% to 44%)
	45-64	5% (3% to 7%)	14% (8% to 22%)	53% (44% to 61%)
Hypertension control	18+	2% (1% to 3%)	9% (5% to 13%)	30% (24% to 36%)
	45-64	2% (1% to 4%)	11% (6% to 16%)	43% (35% to 51%)

Data shown as mean (90% range)

Cardiovascular Disease over the next 50 years under three policies among adults 18+

	Continuation of current policies	Primary care hypertension treatment	Population-level CHW screening
Annual heart attacks	92,393 (56,134 to 135,281)	85,610 (53,187 to 124,496)	67,623 (44,601 to 94,895)
Annual heart attacks averted	NA	6,783 (1,696 to 13,440)	24,770 (11,618 to 41,832)
Annual strokes	94,452 (50,758 to 146,457)	84,696 (47,169 to 131,383)	60,304 (37,032 to 89,923)
Annual strokes averted	NA	9,757 (3,010 to 19,500)	34,148 (13,818 to 60,836)
Annual CVD deaths	159,521 (112,302 to 215,886)	149,250 (104,502 to 198,651)	120,274 (86,363 to 155,725)
Annual CVD deaths averted	NA	10,271 (3,420 to 19,578)	39,247 (19,307 to 62,160)

Data shown as average annual number of events/deaths (90% range). Scaled to estimated size of Ugandan population.

Cost and Cost-Effectiveness over the next 50 years (2024-2074)
Scaled to population size of Uganda

	**Continuation of current policies**	**Primary care hypertension treatment**	**Population-level CHW screening**
DALYs Averted (thousands)	NA	37.8 (-10.8 to 92.5)	147.1 (51.6 to 256.6)
**Cost (millions USD)**			
Screening	NA	NA	17.4 (15.2 to 19.8)
Clinic	7.2 (3.6 to 12.4)	10.1 (5.2 to 16.8)	33.4 (19.9 to 48.4)
Drug	2 (0.8 to 3.7)	7.4 (3.5 to 12.9)	24 (14.5 to 34.7)
CVD	57.5 (14.2 to 141.6)	51.6 (12.7 to 125.9)	37.5 (9.4 to 92.8)
Total	66.7 (21.5 to 154.6)	69.1 (25.9 to 148.1)	112.4 (67.2 to 179.2)
**Incremental cost**	NA	2.4 (-5.9 to 9)	45.7 (18 to 69.9)
Incremental cost-effectiveness ratio (ICER; $USD / DALY averted)*	NA	$62	$388
Net DALYs Averted (thousands) †	NA	36.4 (-18.3 to 94.6)	62.3 (-35.5 to 186.2)
Proportion of setting-scenarios where strategy is cost-effective†	8%	29%	63%

Summary

	**Primary care hypertension Treatment**	**Community-based screening**
Annual Health Benefits	• 7,000 heart attacks averted<br>• 10,000 strokes averted<br>• 10,000 CVD deaths averted	• 25,000 heart attacks averted<br>• 34,000 strokes averted<br>• 39,000 CVD deaths averted
Incremental Annual costs	$2.4 million USD	$46 million USD
Cost-effectiveness in Uganda	Cost-effective in 29% of scenarios	Cost-effective in 63% of scenarios

References

1. World Bank Open Data. World Bank Open Data. Accessed August 31, 2023. https://data.worldbank.org

2. Guwatudde D, Nankya-Mutyoba J, Kalyesubula R, et al. The burden of hypertension in sub-Saharan Africa: a four-country cross sectional study. *BMC Public Health*. 2015;15:1211. doi:10.1186/s12889-015-2546-z

3. Geldsetzer P, Manne-Goehler J, Marcus ME, et al. The state of hypertension care in 44 low-income and middle-income countries: a cross-sectional study of nationally representative individual-level data from 1·1 million adults. *Lancet*. 2019;394(10199):652-662. doi:10.1016/S0140-6736(19)30955-9

4. *Facts on HIV and AIDS in Uganda*. Uganda AIDS Commission; 2021. Accessed July 25, 2023. https://uac.go.ug/media/attachments/2021/09/13/final-2021-hiv-aids-factsheet.pdf
