# Supplementary material for: Cost-effectiveness of leveraging existing HIV primary health systems and community health workers for hypertension screening and treatment in Africa: An individual-based modeling study
Source: PLoS Med. 2025 Jan 24;22(1):e1004531. doi: 10.1371/journal.pmed.1004531 (PMC11805449; doi:10.1371/journal.pmed.1004531)
Supplement: Attachment — Submitted filename: PMEDICINE-D-24-02699R1.pdf [file pmed.1004531.s004.pdf]

Review

Manuscript ID: PMEDICINE-D-24-02699R1

Paper Title: Cost-effectiveness of leveraging existing HIV primary health systems and community health workers for hypertension screening and treatment in Africa: an individual-based modelling study

The paper is overall clearly written, and the subject of the study is important. I have a few comments about the conduct and the presentation of the cost-effectiveness analysis (CEA).

1. The authors are comparing some of the model outputs with epidemiological data, which is very helpful. Given that the model is used to project outcomes over a 50-year period, it would help to also visualize the projected outcomes during this period to ensure that the model's behavior remain reasonable during this long period.
2. The results of the cost-effectiveness analysis (CEA) is mainly presented as "the probability" that an alternative is cost-effective. This is misleading as demonstrated by several recent studies including https://pubmed.ncbi.nlm.nih.gov/17409361/ and https://pubmed.ncbi.nlm.nih.gov/39056310/. Instead, please report the ICER of alternatives or the range of cost-effective thresholds for which each alternative is optimal. It would be also helpful to present the net monetary benefit lines as suggested by above studies.
3. It seems that some of the parameters are not included in the probabilistic sensitivity analysis (i.e., their values are still fixed). Ideally, every parameter with estimated value must be included in the probabilistic analysis to describe the uncertainty in their estimates.
4. In Figure 2 (and when calculating ICER), it is important that the expected overall cost and overall effect of each alternative is calculated based on the results of the probabilistic sensitivity analysis (not based on the deterministic value of each parameter). It would also be helpful to present the clouds of estimated costs and effects in the cost-effectiveness plane in Figure 2. See Chapter 4 in the book "Decision Modelling for Health Economic Evaluation" by Briggs et al.
5. It is stated that "We generated 3000 setting-scenarios to represent a range of settings across eastern, southern, central, and western Africa and to incorporate uncertainty into model outputs." It would help to clarify whether "a scenario" simply refers to a combination of parameter values generated in the probability sensitivity analysis, or scenarios are also defined based on other factors such as assumptions on the model structure.
