# Supplementary material for: Cost-effectiveness of leveraging existing HIV primary health systems and community health workers for hypertension screening and treatment in Africa: An individual-based modeling study
Source: PLoS Med. 2025 Jan 24;22(1):e1004531. doi: 10.1371/journal.pmed.1004531 (PMC11805449; doi:10.1371/journal.pmed.1004531)
Supplement: Attachment — Submitted filename: SEARCH HTN modelling PLOS Med reviewer response 20241109 v3.pdf [file pmed.1004531.s005.pdf]

*Editor/Reviewer comments*

Author responses

We appreciate the time that the Editor and Reviewers have put into their reviews.

----------------------------------------------------------

***Comments from the academic editor:***

*1. I am interested in the rationale for hypertension based screening over CVD risk based population screening based on WHO guidelines (e.g. https://iris.who.int/bitstream/handle/10665/333221/9789240001367-eng.pdf). A risk based strategy is more likely to identify appropriate people for treatment compared with a hypertension strategy - see this paper which suggests a both under- and over-treatment based on risk https://journals.plos.org/plosmedicine/article?id=10.1371/journal.pmed.1003485*

Thanks for this question. The CHW screening approach that we evaluated in this modelling study is designed to achieve high levels of hypertension screening at a population level with simple treatment algorithms that are consistent with guidelines across countries Africa where hypertension treatment thresholds are ≥140/90 mmHg. Anchoring hypertension treatment decisions to blood pressure alone is straightforward for implementers and avoids missed opportunities to treat due to complexity of interpreting treatment algorithms. While absolute benefit will be greater for those with greater baseline CVD risk, we argue that no individual person is "overtreated" when treatment is initiated for blood pressure ≥140/90 mmHg on multiple occasions. Our approach has a strong evidence base in numerous large, randomized trials and meta analyses demonstrating reduction in incident CVD events and mortality with hypertension treatment, including trials showing that a lower target for systolic blood pressure has beneficial effects (SPRINT trial).[1,2]

The Peiris et al PLOS Medicine article is an important contribution to highlight risk-based approaches, though we disagree with thresholds set by that paper for hypertension treatment initiation. There are two major methodologic concerns. First, CVD risk in that study was assessed cross sectionally and pre-treatment blood pressure was only able to be assessed using estimated pre-treatment values. These estimated pre-treatment values assumed uniform treatment response for all individuals (approximately 10 mmHg), ignoring that there is heterogeneity in response and that some individuals may on multiple medications with resulting higher differential between pre-treatment and on-treatment blood pressure. This approach is likely to consider individuals with high pre-treatment blood pressure who had good treatment response to be "overtreated". Second, we have equity concerns about the treatment thresholds proposed. For example, careful review of the non-laboratory based WHO charts for East Africa reveal that the only women in East Africa with a systolic blood pressure of 140-159 mmHg who would be indicated for hypertension treatment are women ≥70 years of age who smoke and have a body mass index ≥35 $kg/m^2$. This would forgo important CVD prevention benefits for a substantial number of people who would stand to benefit from hypertension treatment.

*2. The CHW intervention effects seem overly optimistic- increasing diagnosis rates from 29% to 79% in 45-64 years age group, control rates up to >40% and a RRR of 0.66 for CVD events. The authors state treatment effects are based on SEARCH study results. Could these assumptions be made more explicit in the methods? I note reviewer 3 also sought more information on the implementation model.*

The outputs of our model are driven by input parameters of community-level screening uptake, linkage to care, treatment initiation, and retention in care. We carefully calibrated all of these inputs based on

data from the large multi-country, multi-community SEARCH trial and incorporated both between community variability within SEARCH and potential variability across other settings.

We have added more detailed description of each policy to the methods, based on reviewer comments. We also added a model schematic and enhanced references to the supplement for specific parameters describing probability of antihypertensive medication initiation and retention in care. We are happy to include an additional table in the manuscript text describing these parameters and full justification for the distributions included, if requested by the editor. For now, we have left these in the supplement as even a parsimonious table to describe these parameters currently is more than three pages in length and ultimately leaves out many inter-related details that are best viewed in context with full model parameters provided in the appendix.

The high levels of hypertension diagnosis achieved with the CHW policy are consistent with source data, including recent large-scale CHW hypertension screening data demonstrating that 90% of adults ≥40 years of age can be reached by CHW household-based screening.[3] The new figures requested by Reviewer 4 showing hypertension outcomes over time also highlight that high levels of hypertension diagnosis are not achieved in a single year, but are the cumulative result of annual screening which eventually reaches population saturation. We have added this new series of figures requested by Reviewer 4 to our appendix.

----------------------------------------------------------

*Comments from the reviewers:*

***Reviewer #1:*** *I congratulate the authors on the results of their hard work. This manuscript offers a valuable contribution by evaluating the cost-effectiveness of leveraging HIV health infrastructure and community health workers to tackle hypertension in Africa. The work is well-developed, and the supplemental material is extensive and relevant. I especially appreciate the detailed justification for all the parameter assumptions and the inclusion of the budget impact analysis results. It is also commendable that the manuscript has been drafted in accordance with the Consolidated Health Economic Evaluation Reporting Standards (CHEERS) guidelines.*

We thank the reviewer for these positive comments and their enthusiasm for our manuscript.

*There are just a few comments from my side:*

*-The phrase '3000 setting scenarios' was somewhat confusing. Upon checking the appendix, I found that what is meant appears to be more like '3000 Monte Carlo replications' for probabilistic sensitivity analysis (PSA), where parameters are derived from a given distribution simultaneously. Therefore, it may not be appropriate to call it '3000 setting scenarios'. Alternatively, further explanation could clarify this.*

By ‘setting scenarios’, we refer to a combination of sampled parameter values for a given run of the model. Each model run, with the particular combination of sampled parameter values, is intended to represent variability of national and sub-national settings across the regions as well as uncertainty in model assumptions. These are not Monte Carlo replications as we use different sets of sampled parameter values each time the model is run. We conducted 3000 model runs to account for variability in combinations of parameter values that may exist across different settings. We have added some additional text to the methods to clarify the meaning of setting-scenario in the context of our population-based microsimulation model.

New manuscript text (Methods, Model description, page 4):

A setting-scenario refers to a single run of the model where values for each model parameter are independently and randomly sampled from the set of parameter values. The combined set of model parameters is intended to represent a particular setting (national or sub-national) across settings in eastern, southern, central, and western Africa, as well as representing uncertainty in parameter values over intervention effect sizes.

*-The indexing of the images in the article seems to be incorrect. Please carefully check and revise them.*

Apologies for this oversight. We have corrected the figure references in the text.

*-Abbreviations should be added for 'Figure 1. Hypertension care cascade (2024-2074)'.*

Thank you. We have added definitions of the abbreviations used in this figure.

*-In the methods section, it is stated that the costs were adjusted for inflation to 2023 US dollars, but Figure 2 presents costs in 2024 US dollars. Please ensure consistency.*

We apologize for the oversight. We have corrected the text to indicate that costs were adjusted for inflation to 2024 US dollars.

*Good luck!*

***Reviewer #2:*** *Thank you to the authors for submitting this paper. It is clearly on a very important topic and I really enjoyed reading it. I have a few (relatively minor) comments that I think should be addressed before publication.*

*Methods*

*I know the authors have provided detailed methods and parameters in the appendix, however I would like some of this brought into the main text. The reality is that not many readers will venture into the appendices to look at the methods, and as this is (for me) the most important part of the paper I would like it to be expanded. Specific things I would bring into the main are a model schematic and table with some of the key variables and their sources.*

Thank you for this point. We understand and agree with bringing more detail into the main text. We continue to refer interested readers to the supplement for detailed review of all relevant parameters which will provide a more expansive view of the numerous interrelated factors which we considered in the model. We have now added a model schematic in the main text. Though this schematic somewhat over-simplifies the model, it does provide a conceptual overview of how individuals move through relevant states regarding hypertension treatment within the model. We have not added an additional table of model parameters into the main text, though can do so if desired by the Editor. We constructed

such a table, however it is nearly 3 pages in length and ultimately leaves out many inter-related details that are best viewed in context with full model parameters provided in the appendix.

New Figure (Figure 1):

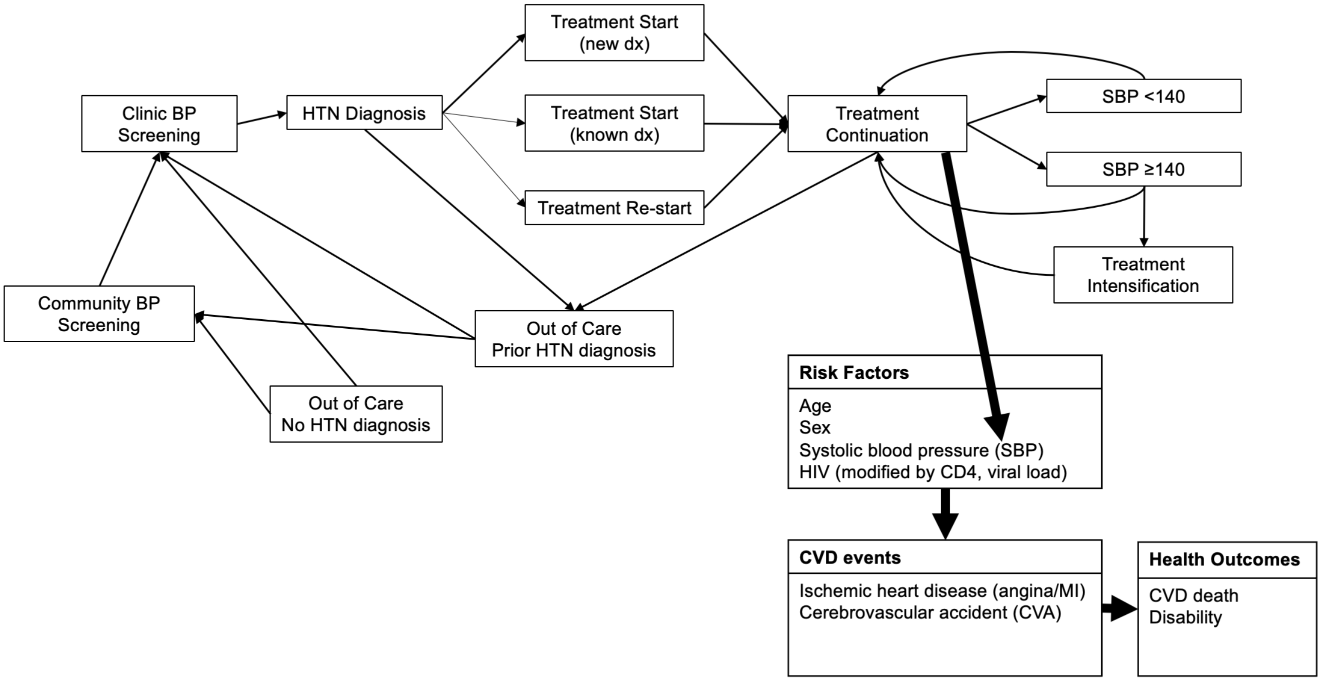


Note: Here, we provide this simplified model schematic to provide a broad overview of how individual persons in the model move through the continuum of hypertension care over time and how hypertension treatment impacts CVD outcomes. This is an individual model where variables for each individual are updated each 3-month time step. The full individual model is defined by the set of variables and the updating rules which can be seen in full in the Appendix.

*Policy Comparison*

*The authors compare the intervention to two other policies. I was wondering if the authors could comment on whether there are any other relevant comparators that could have been included? I appreciate that given the limitations in the available data it may not have been possible to include them however an acknowledgment of any excluded relevant comparators would be useful (if applicable). For me this is especially relevant given the heterogeneity in the African countries included in the analysis. I note that the authors include a discussion of the fact that the most relevant comparator will change in the future years.*

Our analysis compares two interventions (policies) to the current state of hypertension care across African countries, accounting for variability in current uptake of hypertension diagnosis and screening. We attempted to capture this current state of hypertension treatment as best as possible, based on available data and believe this to be the most relevant comparator for the two policy interventions we included in our analysis. We chose the two policies we evaluate in this analysis because they were feasible in large-scale studies, we have population-level data to inform modeling parameters, and because of their potential for population-level improvements in health outcomes. We also acknowledge, as pointed out by the reviewer, that other potential policies could be considered and that we are unable to assess all potential policies that could be implemented. One strength of our model is that it is well set-up to incorporate additional policies for consideration, should new data arise to suggest additional promising approaches to cardiovascular disease prevention in this setting. We have added an additional

statement highlighting the limitation of not being able to consider all potential alternative policies in our analysis.

New text (Discussion, limitations paragraph, page 19)
An additional limitation is that we were not able to include all potential policy alternatives in our analysis. We selected two policies for consideration based on available data in the literature to suggest that these approaches may have substantial impact on population-level CVD prevention. In the future, our model is well positioned to incorporate additional policies as new data arises regarding the most impactful and efficient approaches to CVD prevention.

*Cost Effectiveness Analysis*

*Could the authors please further justify the use of the "$500 per DALY averted" threshold in the base case? I note in Figure 3 this is referred to as the upper bound of the HIV threshold*

We have added additional justification for this threshold. We have removed reference to HIV in the figure, as this threshold more closely approximates domestic funding in lower-middle income settings and donor-funding in lower income settings.

Revised text (Methods: Cost-effectiveness analysis, page 8):
Cost-effectiveness thresholds are uncertain; though a threshold of $500 USD which approximates the threshold estimated for lower-middle income countries (e.g. Kenya) and better accounts for opportunity costs than much higher previously used CETs of 1-3 times GDP.[4–6] In lower-income settings, a cost-effectiveness threshold of $500 per DALY averted presumes at least some degree of donor funding; we also conducted sensitivity analysis to evaluate cost-effectiveness under varying cost-effectiveness thresholds to consider scenarios where funding is provided by domestic sources in lower income settings.[4]

***Reviewer #3:*** *In general this is a very thorough manuscript on the impact and cost-effectiveness of leveraging existing HIV-related healthcare infrastructure to decrease the burden of hypertension. I do, however, have a few significant points for consideration-*

*1. My biggest point of interest is around the 'how' of implementation and what these interventions precisely mean. The difference in these three interventions, I assume, ultimately come down to differences in uptake/care seeking if hypertension care is provided in different ways, and retention in care after the initial diagnosis. If this is the case, there needs to be a better description of what the SOC, CCC and CHW are and what they effectively mean in practice. What is the SOC, how do people typically seek care now, when would someone be offered to be screened? In practice for CCC, what would change, how would that mechanistically alter who receives care? A proper description in this main text of this manuscript without needing to refer to the SEARCH study is needed for all three scenarios.*

Thank you for this important point regarding the how of implementation. We acknowledge that implementation may differ across settings and across policies, and we have incorporated variability in numerous model parameters defining uptake of screening, linkage to clinic, treatment initiation, treatment intensification, and retention in care to incorporate variability in implementation and resulting effect on outcomes. We presume that typical SOC, in most settings, involves hypertension screening and treatment delivered in district/regional hospital-based hypertension/non-communicable disease clinics. While SOC will certainly vary from setting to setting, it is clear from current literature

across the region that uptake of hypertension diagnosis, treatment, and resulting hypertension control is quite low. We further calibrated SOC parameters against Global Burden of Disease data and other literature to mirror best available estimates of hypertension diagnosis, treatment, and control. An important driver of low levels of hypertension diagnosis, treatment, and control is current lack of funding for hypertension and other noncommunicable disease care. Our analysis highlights potential health impacts that could be achieved with increased investment. In our model, the CCC policy entails integration of hypertension screening and treatment within primary care clinics. We assume in most cases that this involves integration into the same systems that deliver HIV care, though also allow for variation in that some hypertension care may not be fully integrated but will still occur within the context of primary care. Model parameters for both implementation and cost of this model are derived largely from the SEARCH study and the INTE-AFRICA studies in Kenya, Uganda, and Tanzania. The CHW policy entails household-based hypertension screening with referral to clinic for diagnosis and treatment. We have added an additional paragraph to the methods to expand on each of these policies.

New text (Methods: Policy Comparison, page 5-6):
The SOC policy represents the current state of hypertension care delivery, which is often only available in centralized hospital-based hypertension clinics that are inaccessible to many people due to long transportation times, high out-of-pocket costs, and competing priorities.[7] The CCC policy entails integration of hypertension screening and treatment within primary care clinics, as conceptualized in both the SEARCH and INTE-AFRICA studies and including hypertension care delivery for both people with and without HIV.[8–10] Key strategies in these studies include provider training on patient-centered care/friendly services and hypertension management, changes in clinic workflows to incorporate routine blood pressure screening and nursing triage, and adoption of clinic record systems that mirrored those used for long-term monitoring of HIV (e.g. patient cards to record blood pressure and medications over multiple visits). Such interventions were low cost but effective for improving blood pressure treatment and control compared to non-integrated care.[10,11] In both SOC and CCC policies, we presume that blood pressure screening is only conducted within clinic-based settings; we further presume that the CCC model does not increase rates of presenting to clinic for hypertension screening. The CHW policy represents a frameshift by implementing hypertension screening in the community, by community health workers. We presume that screening is conducted at the household level, alongside other CHW-based household interventions. For each of these policies, we modeled individual-level effects on hypertension screening, diagnosis, and treatment over time based on data from the SEARCH studies (Appendix Table S4-S6, S8).[3,10,12]

2. *For the CHW scenario- is this community testing? Door to door? Assumptions regarding the 'how' will alter effectiveness of the intervention and who is ultimately tested. If this is door to door, what is assumed about how many people a CHW can reach per day? What proportion are rural vs urban (in some settings). If it's door to door, what transport costs are being assumed (as community-level screening is typically extraordinarily costly due to things like transport and how spread-out people are in some settings, and the cost of general logistics and planning). In the supplemental material, I see that the cost estimates also come from SEARCH, but how 'transportable' are those estimates to a 'general' population across sub-Saharan Africa? (If costs of implementation varied greatly by things such as geography, population density, etc., then the costs should be weighted to reflect an 'average' population across the region) Also, what exactly was included in that $3 per person screened?*

In the CHW scenario, we presume this is household-based (door-to-door) screening and have now added this to the new paragraph in the Methods section, described above in point #1. As detailed in Table S9 of the Appendix, we assume that each CHW is responsible for approximately 100 households,

including approximately 75 adults ≥40 years of age. We conservatively assume that CHWs would screen 1-2 people per working day over a period of 2 months, with 50% of CHW salary allocated to hypertension activities. This rate of screening would allow screening to fit in with other existing community health worker responsibilities. Alternative approaches to implementation could be considered for similar costs (e.g. extending screening over a longer period of time at lower effort allocated to hypertension screening). Because community health workers reside in the communities where they work, transportation costs are minimal to move between households and effort to travel to individual households would be allocated across multiple other activities (e.g. maternal child health interventions, malaria prevention/treatment, etc). We further detail the costs included in the $3 per person screened in Table S9, which include blood pressure cuffs, batteries, training, supervision, and overhead/administrative costs. We acknowledge that these costs may vary by region (as we highlight in Table S10 with analysis of minimum wage policies across countries and settings (i.e. rural/urban) in Eastern, Southern, Central, and Western Africa. To accommodate alternative cost scenarios in our model, we also conduct sensitivity analysis under conditions where costs are 50% lower and 200% higher than base-case costs.

*3. Are there any other costs that would need to be included when moving from SOC to CCC? Such as the cost of integrating information systems, renovations to clinics?*

Yes, we do incorporate additional costs incurred when moving from SOC to CCC (and CHW) policies. We incorporate additional annual implementation costs which would include additional training for clinicians and clinic staff (e.g. hypertension care delivery, integrated care, and use of information systems for chronic hypertension management). These costs are in addition to existing continuing medical education and clinic supervision costs. We assume that clinics will use the same physical and information system infrastructure, so no renovation costs are incorporated.

*4. Within the SEARCH study, were there population- or implementation-specific factors that increased or decreased the uptake of initial diagnosis? Was any of that granularity used to refine the modelled results (e.g. were gender-specific estimates, or rural/urban-specific estimates used from the SEARCH study within the agent-based model?)?*

Thanks for this question. Yes, there was some heterogeneity between communities and we used this to inform the range of estimates included in many hypertension treatment initiation and retention parameters, as detailed in the Appendix. We also incorporated observations in SEARCH and elsewhere in the literature that women are more likely to engage in healthcare interventions than men. In the model, this is incorporated in our parameter of being 'hard to reach', which is the proportion of men and women in a given setting-scenario that will not undergo hypertension screening in the community or clinic. We model this separately for women and for men, incorporating higher likelihood that men are hard to reach compared to women. We include detailed ranges across communities and, where observed, by gender throughout the Appendix.

*5. Are there additional scenarios that can be explored, or other types of interventions that could be assessed that could increase uptake in care? (e.g. integrated care + demand generation, integrated care + targeted CHW outreach (say, in urban areas, or in areas with higher prevalence of hypertension), integrated care + mobile apps, screening integrated into the OPD, etc) Without exhausting all potential competing alternatives, it would be difficult to say whether the two interventions evaluated in this study are consistently on the cost-effectiveness frontier or not.*

This is an excellent point. We agree that there may be other types of interventions that could be assessed. We chose two policies that had strong evidence for feasibility and effectiveness in large population-level studies. We were unable to assess all potential alternatives though did attempt to include two representative incremental policies that could be considered for adoption. One strength of our model is that it is well set-up to incorporate additional policies for consideration, should new data arise to suggest additional promising approaches to cardiovascular disease prevention in this setting. As described in response to Reviewer #2, we have added an additional statement highlighting the limitation of not being able to consider all potential alternative policies in our analysis.

New text (Discussion, limitations paragraph, page 19):
An additional limitation is that we were not able to include all potential policy alternatives in our analysis. We selected two policies for consideration based on available data in the literature to suggest that these approaches may have substantial impact on population-level CVD prevention. In the future, our model is well positioned to incorporate additional policies as new data arises regarding the most impactful and efficient approaches to CVD prevention.

***Reviewer #4:*** *The paper is overall clearly written, and the subject of the study is important. I have a few comments about the conduct and the presentation of the cost-effectiveness analysis (CEA).*

*1. The authors are comparing some of the model outputs with epidemiological data, which is very helpful. Given that the model is used to project outcomes over a 50-year period, it would help to also visualize the projected outcomes during this period to ensure that the model's behavior remain reasonable during this long period.*

Thank you for this suggestion and we are glad that the reviewer found our comparison of model outputs to epidemiologic data to be helpful. We have generated additional plots to visualize changes in hypertension prevalence, diagnosis, current treatment, and hypertension control (defined as SBP <140 mmHg in the model). We have added these graphics here for reference and have also added them to Section 5 the Appendix (section with expanded results). These graphs highlight slight increases in hypertension prevalence over time due to better survival with the CCC and CHW policies. We note that the CHW policy includes annual screening, which results in an annual increase in diagnosis, treatment initiation, and hypertension control. In our model, probability of return is lowest following the first visit, therefore drop-out from care is steeper among those newly engaging in care (compared to individuals who have established a more stable pattern of engagement).

New supplemental figures (Appendix, Section 5):

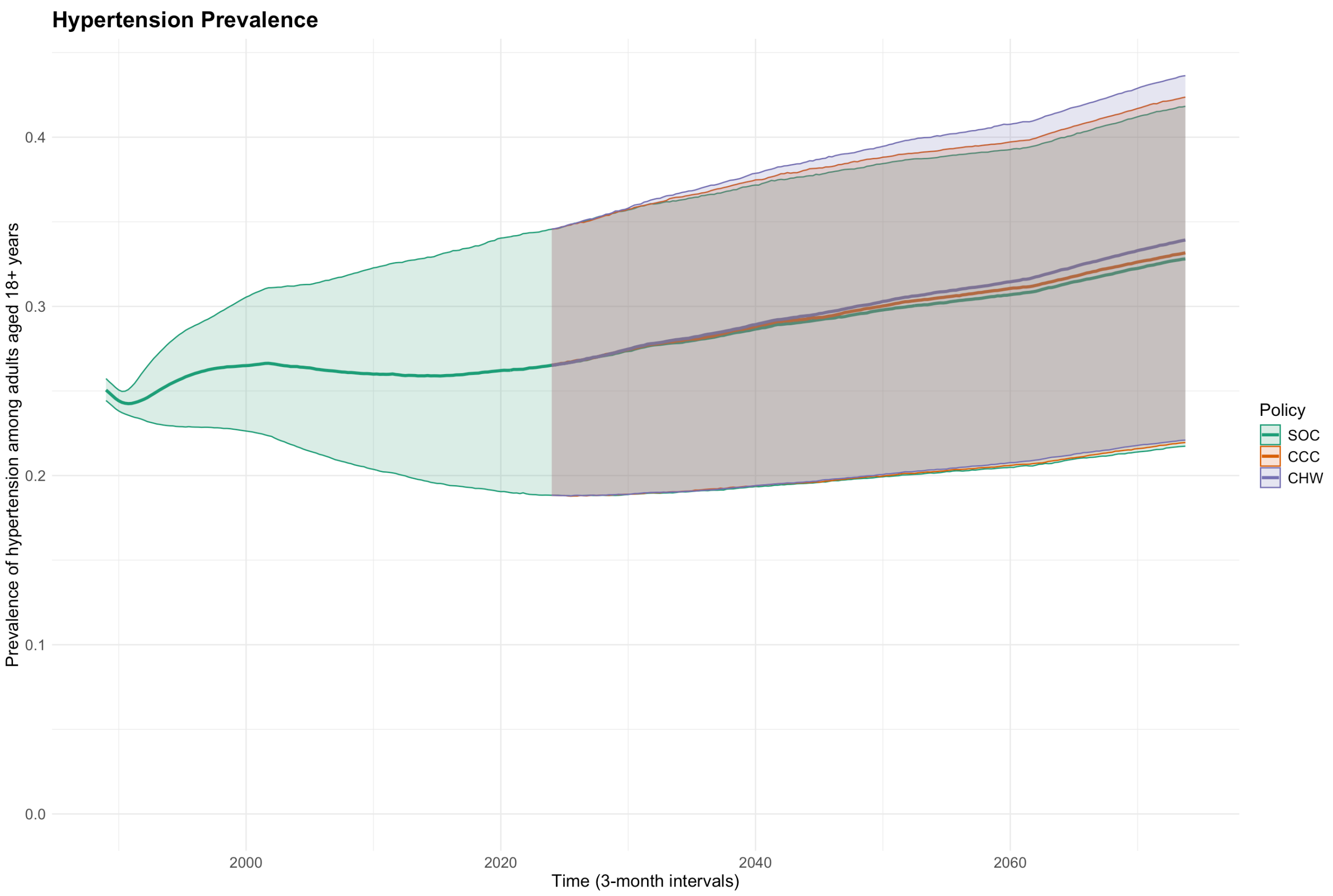
Hypertension Prevalence
Prevalence of hypertension among adults aged 18+ years
0.0
0.1
0.2
0.3
0.4
2000
2020
2040
2060
Time (3-month intervals)
Policy
SOC
CCC
CHW

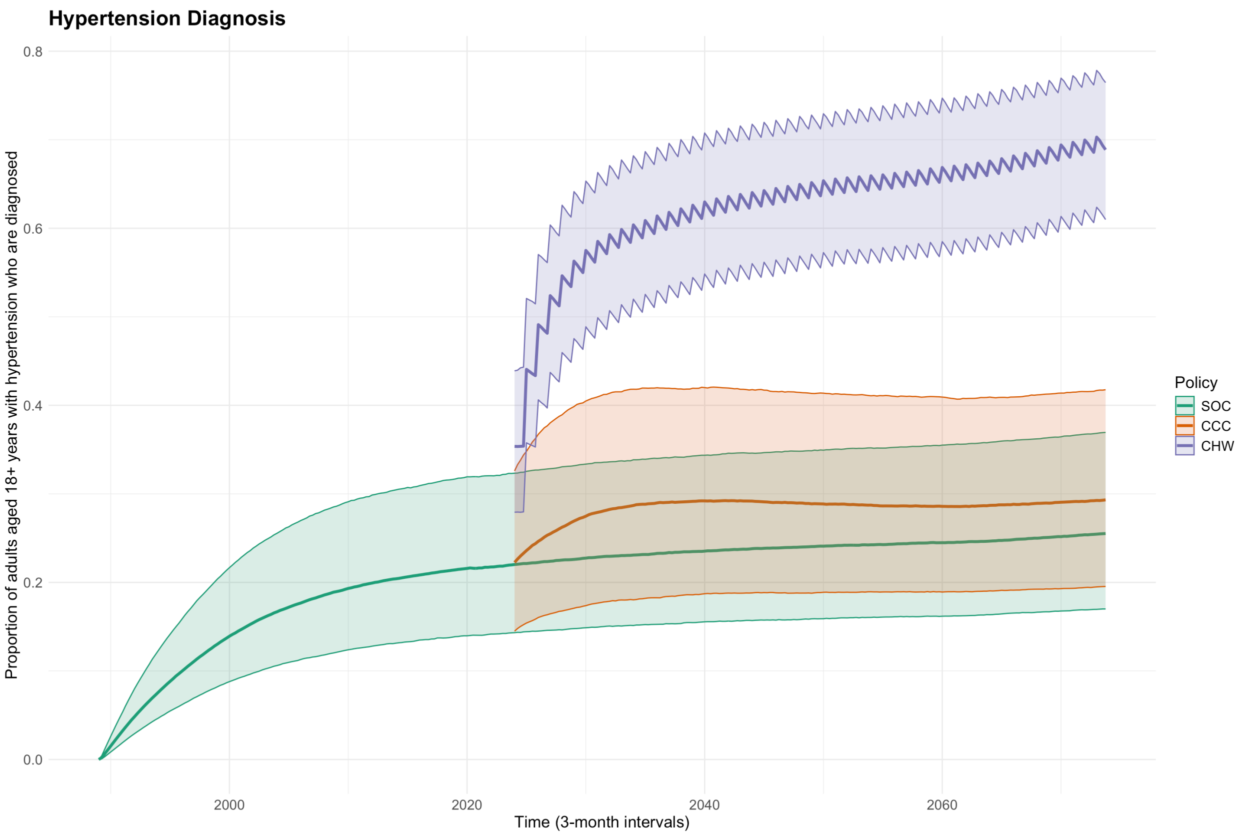
Hypertension Diagnosis
Proportion of adults aged 18+ years with hypertension who are diagnosed
0.0
0.2
0.4
0.6
0.8
2000
2020
2040
2060
Time (3-month intervals)
Policy
SOC
CCC
CHW

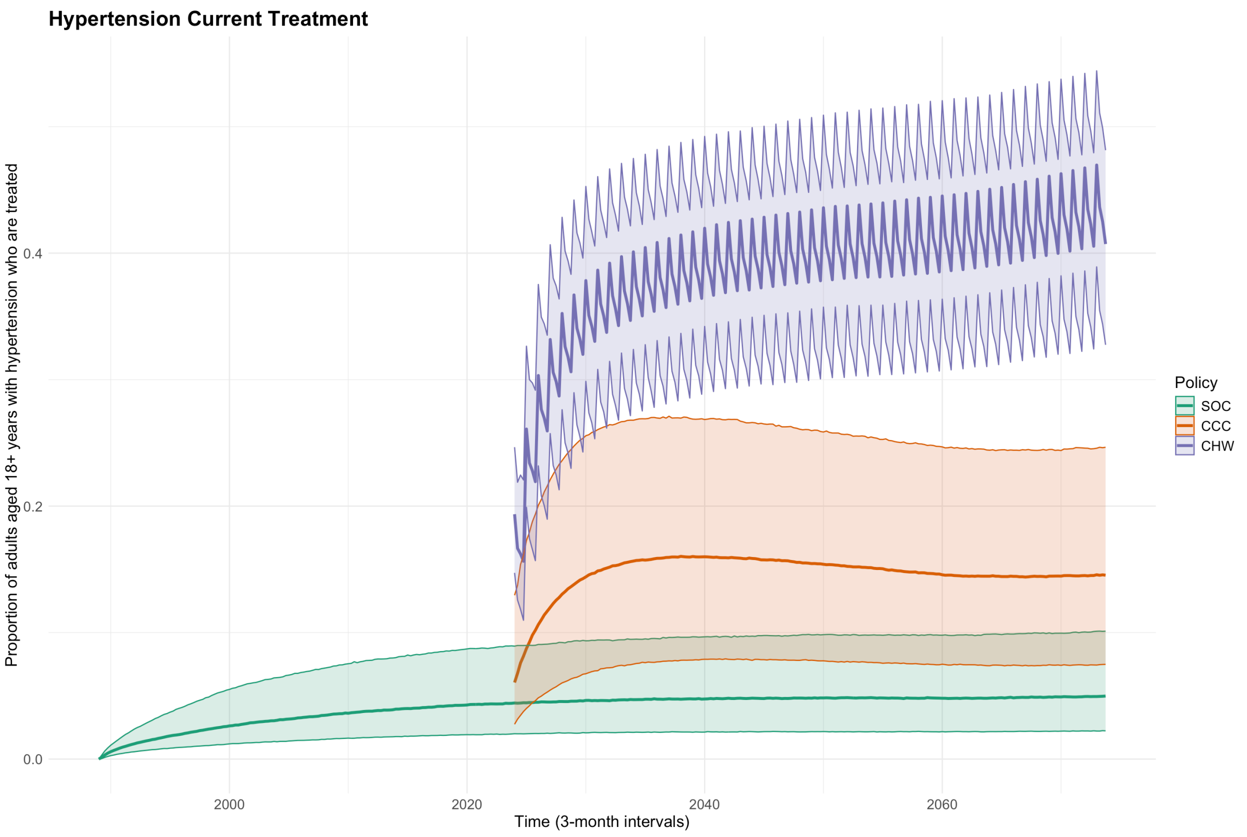
Hypertension Current Treatment
Proportion of adults aged 18+ years with hypertension who are treated
0.0
0.2
0.4
2000
2020
2040
2060
Time (3-month intervals)
Policy
SOC
CCC
CHW

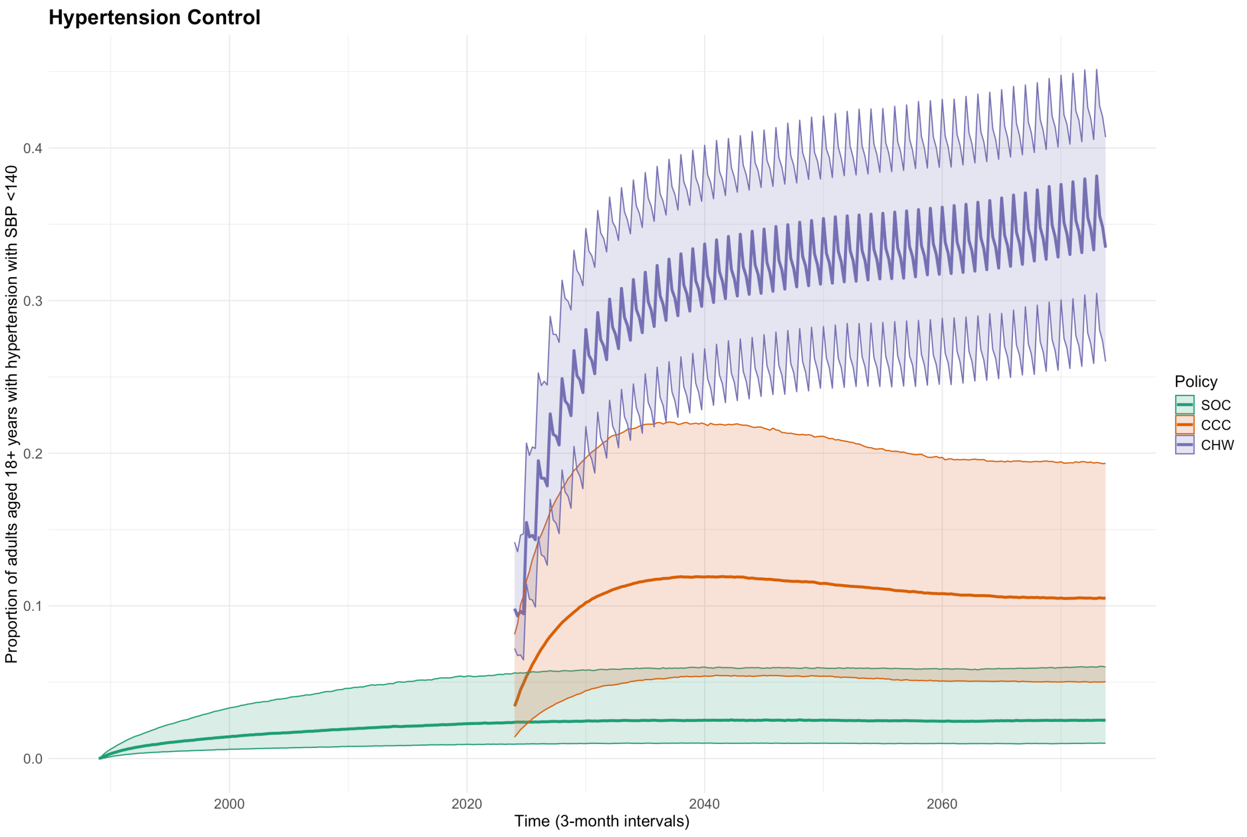
Hypertension Control
Proportion of adults aged 18+ years with hypertension with SBP <140
0.0
0.1
0.2
0.3
0.4
2000
2020
2040
2060
Time (3-month intervals)
Policy
SOC
CCC
CHW

*2. The results of the cost-effectiveness analysis (CEA) is mainly presented as "the probability" that an alternative is cost-effective. This is misleading as demonstrated by several recent studies including https://pubmed.ncbi.nlm.nih.gov/17409361/ and https://pubmed.ncbi.nlm.nih.gov/39056310/. Instead, please report the ICER of alternatives or the range of cost-effective thresholds for which each alternative is optimal. It would be also helpful to present the net monetary benefit lines as suggested by above studies.*

Thank you for this point and for the citations. We agree with the limitations of cost-effectiveness acceptability curves outlined in the Koerkamp et al article provided by the reviewer, namely that reporting the probability that a given policy is cost-effective at each cost-effectiveness threshold does not provide any information for how much better – or worse – the alternatives may be. We do provide information on the magnitude of health benefit using net DALYS (Table 4), reporting the mean and 90% range of health benefits (DALYs averted), costs, and net DALYs across all setting scenarios, which provides insight into the range of potential outcomes. In response to this comment, we have now added a new Figure (Figure 4B) that demonstrates the range of net DALYs across cost-effectiveness thresholds, as suggested in the Koerkamp et al paper. It is apparent from this figure that for very low cost-effectiveness thresholds approaching $100 USD/DALY averted, such as the domestic threshold for health interventions in some low-income African countries, the CHW policy is associated with a large negative number of net DALYs averted – suggesting large opportunity costs for implementing the CHW policy in settings with a very low cost-effectiveness threshold. As cost-effectiveness thresholds increase to the range of $300-500 USD/DALY averted (estimated level in some lower and lower-middle income countries, or donor funded initiatives in lower income countries – such as HIV treatment), the range of potential net DALYs is narrower between different policies. We do think the cost-effectiveness acceptability curve (now Figure 4A) provides complimentary information regarding the proportion of setting scenarios where each policy is cost-effective, particularly at cost-effectiveness thresholds where there is considerable overlap in 90% ranges of net DALYs averted across setting-scenarios (due to both differences between settings and uncertainty in parameter values for a given setting).

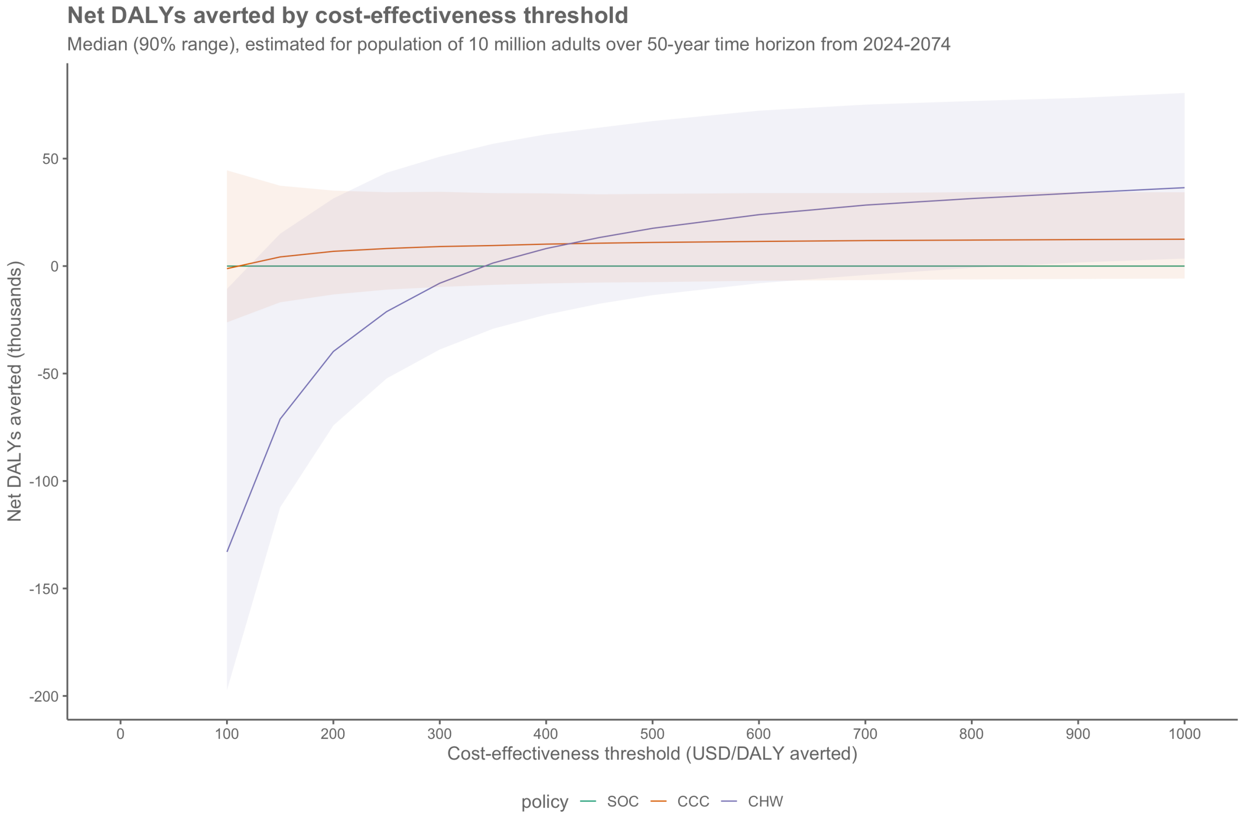

Net monetary benefit is a complimentary measure to Net Health Benefit, which we report in our analysis using net DALYs. Because we already report net DALYs, we do not think that it would add additional information to also report net monetary benefit.

3. *It seems that some of the parameters are not included in the probabilistic sensitivity analysis (i.e., their values are still fixed). Ideally, every parameter with estimated value must be included in the probabilistic analysis to describe the uncertainty in their estimates.*

Our sensitivity analysis (Table 5) did not fix values of other parameters in the model. In Table 5, we simply stratify all model runs by strata of the variables listed in the first column. We have added a sentence to the methods to describe the approach to Table 5.

New text (Methods: Cost-effectiveness analysis, page 8):
To understand the impact of baseline setting-scenario characteristics, we also report the proportion of settings where each policy is the cost-effective choice among strata defined by selected baseline setting-scenario characteristics.

4. *In Figure 2 (and when calculating ICER), it is important that the expected overall cost and overall effect of each alternative is calculated based on the results of the probabilistic sensitivity analysis (not based on the deterministic value of each parameter). It would also be helpful to present the clouds of estimated costs and effects in the cost-effectiveness plane in Figure 2. See Chapter 4 in the book "Decision Modelling for Health Economic Evaluation" by Briggs et al.*

We have updated Figure 2 (now Figure 3 due to addition of model schematic) to include the suggested distribution of estimated costs and effects using a scatterplot of individual setting-scenario data points. We added reference to this additional analysis in the revised manuscript text

Revised text (Methods: Cost-effectiveness analysis, page 8):

We plot the overall ICER for each policy, along with individual DALYs averted per cost for each of the 3000 setting-scenarios generated by the model.

**Figure 3:**

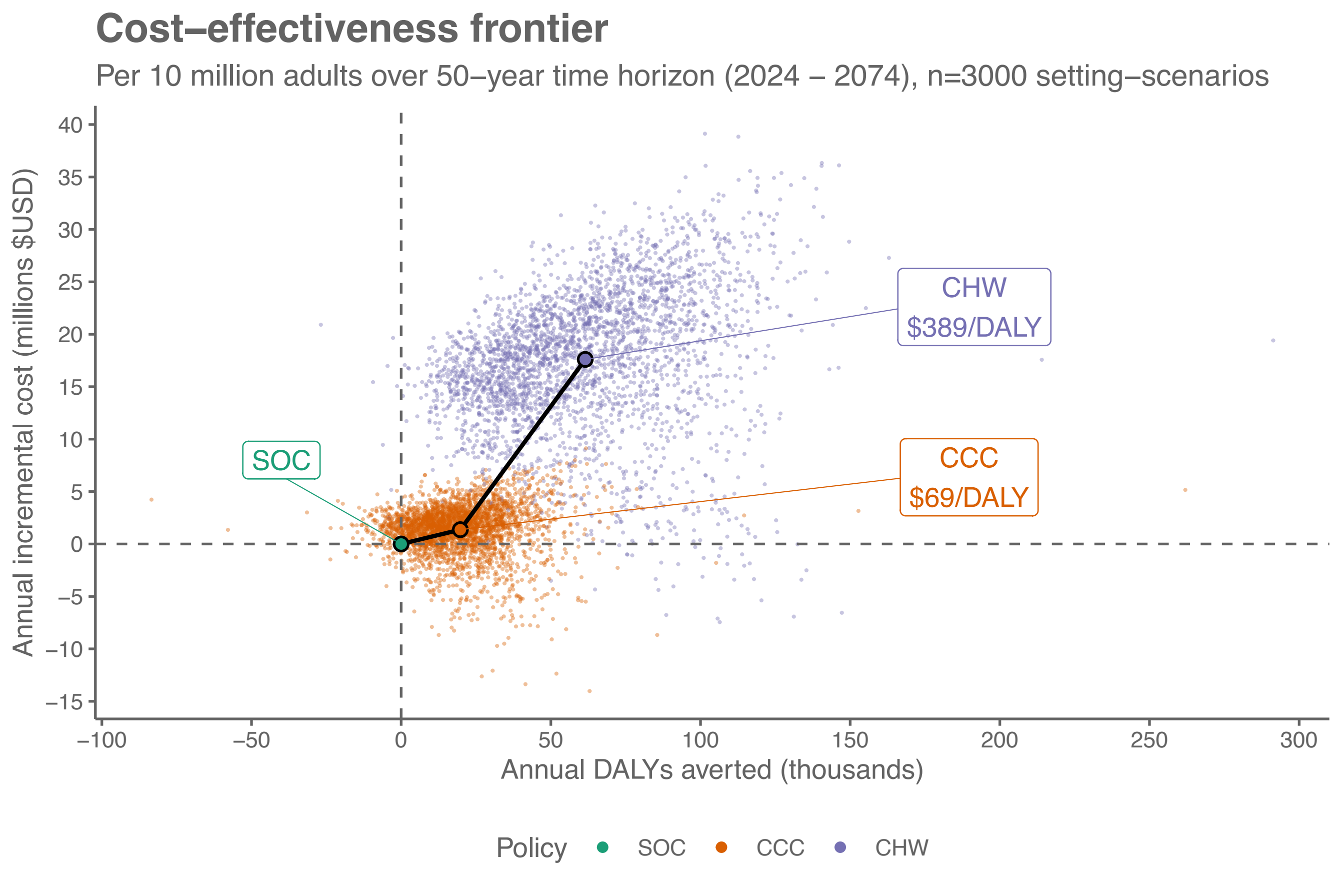


5. *It is stated that "We generated 3000 setting-scenarios to represent a range of settings across eastern, southern, central, and western Africa and to incorporate uncertainty into model outputs." It would help to clarify whether "a scenario" simply refers to a combination of parameter values generated in the probability sensitivity analysis, or scenarios are also defined based on other factors such as assumptions on the model structure.*

A setting-scenario refers to a single run of the model where values for each model parameter are independently and randomly sampled from the set of parameter values. The combined set of model parameters is intended to represent a particular setting (national or sub-national) across settings in eastern, southern, central, and western Africa. We have added this additional clarification to the manuscript text

<u>New manuscript text (Methods, Model description, page 4):</u>
A setting-scenario refers to a single run of the model where values for each model parameter are independently and randomly sampled from the set of parameter values. The combined set of model parameters is intended to represent a particular setting (national or sub-national) across settings in eastern, southern, central, and western Africa.

*Reviewer #5 (additional review received after decision letter):*

· *Assumptions of efficacy may be overly optimistic, as efficacy estimates from randomized trials are often not matched in longer-term population-based / pragmatic studies.*

There is very strong evidence for the efficacy of antihypertensive therapy for reducing cardiovascular disease outcomes, and we have used estimates from large meta-analyses of randomized trials conducted over long time frames in our model to estimate the effects of antihypertensive therapy when taken as intended. The ultimate effectiveness of antihypertensive therapy will depend on how extensively antihypertensives are deployed. Our model includes a range of assumptions across all modelling parameters to capture implementation ranging from levels seen in large-scale pragmatic trials, to lower uptake that approximates settings where implementation is less successful. We include in our assumptions the possibility of lower uptake of interventions in real-world implementation. For example, for CHW population level screening, a recent large-scale trial using Ministry of Health community health workers in nine communities in Kenya and Uganda achieved approximately 90% hypertension screening coverage at baseline and again after 1 year,[3] though we assume in our model lower uptake of approximately 65% screening coverage among men and 70% among women with ranges around these parameters. One important role that this model can play in policy decisions is to highlight a range of outcomes under differing implementation characteristics. We have added additional discussion of uncertainty of true implementation in real-world settings.

Revised text (Discussion, limitations section, page 17):
Implementation of other aspects of these policies may also vary between policies and across setting-scenario. We therefore include a wide range of potential screening uptake in our modelling parameters to address uncertainty both in policy effects and implementation across settings

· *The long time frame (50 years) favors C-E of prevention interventions over treatment interventions, it (and the effect of alternative discount rates) should be evaluated in sensitivity analysis. Few policies these days are made with a 50-year time horizon.*

We modelled a 50-year time horizon, to capture the long-term effects on health outcomes and costs from the policies, over expected life horizons. Of course, discounting means that as we project further into the future, differences between the alternatives have a limited effect on cost-effectiveness assessed in present value terms. To address this comment, we have added a sensitivity analysis using a 5% discount rate for both costs and health effects. In sensitivity analysis with this higher 5% discount rate, which reduces the impact of costs and health effects that occur in the more distant future, the ICER for the CHW policy increased to $444 USD/DALY averted and the proportion of setting-scenarios where the CHW policy was cost-effective was reduced to 51%. This sensitivity analysis demonstrates that the addition of CHW population level screening for all adults aged 40 years and older is expected to be cost-effective in half of settings even when outcomes and costs in the more distant future are given less weight.

· *Demographic model*

*o “Each run of the simulation program creates 100,000 simulated people who will be age 15 or above at some point between 1989 and 2071” The rationale for the year / age parameters is not clear.*

This is the structure of the HIV Synthesis model, which has been used as the basis of numerous other analyses[13–15] and which we used for our analysis. The HIV Synthesis model generates a simulated population in 1989, which is the presumed start of the HIV pandemic in the model. The model includes adults aged 15 years and older because this mirrors most HIV incidence and prevalence estimates in the literature; however, persons in the model are only eligible for hypertension screening and treatment once they are 18 years or older and the CHW screening policy only focuses on adults aged 40 years and older for community-based screening. Model behavior prior to the start of this study (2024) do not

impact our analysis and are intended to model the evolution of population demographics and the HIV pandemic. In Table 1, we provide model estimates for HIV and hypertension alongside estimates from the literature, demonstrating that the model is well calibrated to the contemporary data from the literature at the time of analysis start.

- *The reasons for the use of 1997 mortality data (pre HIV mortality) is not clear.*

We have added clarification for this data to the supplement (Appendix, page 4). We use 1997 mortality data from South Africa to inform non-HIV, non-TB, and non-CVD mortality rates in the early portion of the model (prior to 2019). We use this data source because South Africa has more comprehensive mortality data than most settings in East, Southern, Central and West Africa and this year is prior to the significant impact of HIV-related deaths on mortality overall estimates. While these estimates form the basis of mortality estimates in the early portion of the model, as time progresses in the model, non-CVD, non-HIV, and non-TB age-specific mortality rates are updated to those from most recently available Global Burden of Disease estimates in 2019. The 2019 GBD data is the only data that informs the present analysis.

- *Hypertension model*

- *Probability of retention in the model does not seem consistent with the source data.*

Model parameters were estimated based on retention in treatment over a three period in SEARCH following linkage to care after community screening. Probability of retention in hypertension care based on intervention arm of SEARCH study (mirroring CCC policy) showed that 48% remained engaged in hypertension care after 3 years.[10] In the model, we assumed a 5% rate of re-engagement in care in each quarter, and sampled from parameters for probability of return for a follow-up visit that resulted in average retention in care after 3 years of 49% (range 39-55%). Our parameters assumed that likelihood of return for a follow-up visit was lower after the initial visit and increased the longer someone remained in care.

The proportion of persons with hypertension in the model who are on hypertension treatment at any given time is influenced by retention parameters, as well as parameters related to likelihood of diagnosis. In the CCC policy, the proportion on antihypertensive therapy at any given time was 15% (90% range 7.4 to 25%). This proportion on treatment is lower than our retention estimates because many persons with hypertension did not have a known diagnosis. In the CHW policy, the proportion on treatment is higher because the CHW policy included annual screening, resulting in increased re-engagement in care each year. This re-engagement in care (and steeper drop-out from care immediately after (re-)linkage) can be seen in the above graphs included in our response to Reviewer 4 (point #1).

- *The perspective – that of the health system – excludes an important component, the time cost associated with screening. The benefit measures is individual-focused, I believe the cost measure should include the (time) cost borne by the individual. (Alternatively, this could be incorporated into the health system perspective, e.g., to ensure adherence to regular screening, it may be necessary to incentivize individuals, the incentive would represent the opportunity cost to the individual)*

Our objective was to inform budget allocation, assuming the principal objective of improving population health, so this has informed our choice of perspective. Whilst we agree that patient-facing costs might

also be important to consider, not least because they might affect the demand for care, we have not considered them in the present analysis. We have included a range of estimates, including lower uptake of hypertension interventions that could reflect lower demand for these services in some settings due to patient-facing opportunity costs. We did not consider additional demand-generating policies such as incentivization in this analysis.

· *Supplement 4: Results from the Uganda example seem inconsistent: with a lower ICER of $62/DALY averted (compared to $388 for CHW screening) one would expect a larger percentage of setting scenarios to be cost-effective (currently 29% vs. 62%). The same conclusion holds when looking at the relative benefits and costs in the blue summary figure below.*

The ICER is calculated from the average incremental cost and average incremental health effects (compared to the next most effective policy), whereas the proportion of setting scenarios is determined by calculating net DALYs averted for each individual setting scenario. In each given setting scenario, the policy that led to the greatest number of net DALYs averted was considered cost-effective. In the Uganda example, the mean ICER values decreased slightly, whereas the proportion of settings where neither CCC or CHW policy was cost-effective increased slightly (from 7% to 8%).

**References**


1. SPRINT Research Group, Wright JT, Williamson JD, et al. A Randomized Trial of Intensive versus Standard Blood-Pressure Control. *N Engl J Med*. 2015;373(22):2103-2116. doi:10.1056/NEJMoa1511939

2. SPRINT Research Group, Lewis CE, Fine LJ, et al. Final Report of a Trial of Intensive versus Standard Blood-Pressure Control. *N Engl J Med*. 2021;384(20):1921-1930. doi:10.1056/NEJMoa1901281

3. Hickey M, Owaraganise A, Ogachi S, et al. Feasibility and early outcomes of the SEARCH multi-disease population-level community health worker-led intervention for integrated HIV and severe hypertension services in rural East Africa. Poster presented at: International AIDS Society; July 24, 2024; Munich, Germany.

4. Woods B, Revill P, Sculpher M, Claxton K. Country-Level Cost-Effectiveness Thresholds: Initial Estimates and the Need for Further Research. *Value Health J Int Soc Pharmacoeconomics Outcomes Res*. 2016;19(8):929-935. doi:10.1016/j.jval.2016.02.017

5. Ochalek J, Lomas J, Claxton K. Estimating health opportunity costs in low-income and middle-income countries: a novel approach and evidence from cross-country data. *BMJ Glob Health*. 2018;3(6):e000964. doi:10.1136/bmjgh-2018-000964

6. Vallejo-Torres L, Claxton K, Edney LC, et al. Challenges of calculating cost-effectiveness thresholds. *Lancet Glob Health*. 2023;11(10):e1508. doi:10.1016/S2214-109X(23)00351-0

7. Brathwaite R, Hutchinson E, McKee M, Palafox B, Balabanova D. The Long and Winding Road: A Systematic Literature Review Conceptualising Pathways for Hypertension Care and Control in Low- and Middle-Income Countries. *Int J Health Policy Manag*. Published online July 18, 2020. doi:10.34172/ijhpm.2020.105

8. Van Hout MC, Zalwango F, Akugizibwe M, et al. Implementing integrated care clinics for HIV-infection, diabetes and hypertension in Uganda (INTE-AFRICA): process evaluation of a cluster randomised controlled trial. *BMC Health Serv Res*. 2023;23(1):570. doi:10.1186/s12913-023-09534-0

9. Kivuyo S, Birungi J, Okebe J, et al. Integrated management of HIV, diabetes, and hypertension in sub-Saharan Africa (INTE-AFRICA): a pragmatic cluster-randomised, controlled trial. *The Lancet*. 2023;402(10409):1241-1250. doi:10.1016/S0140-6736(23)01573-8

10. Hickey MD, Ayieko J, Owaraganise A, et al. Effect of a patient-centered hypertension delivery strategy on all-cause mortality: Secondary analysis of SEARCH, a community-randomized trial in rural Kenya and Uganda. *PLoS Med*. 2021;18(9):e1003803. doi:10.1371/journal.pmed.1003803

11. Shade SB, Osmand T, Kwarisiima D, et al. Costs of integrating hypertension care into HIV care in rural East African clinics. *AIDS*. 2021;35(6):911-919. doi:10.1097/QAD.0000000000002834

12. Hickey MD, Owaraganise A, Sang N, et al. Effect of a one-time financial incentive on linkage to chronic hypertension care in Kenya and Uganda: A randomized controlled trial. *PloS One*. 2022;17(11):e0277312. doi:10.1371/journal.pone.0277312

13. Phillips AN, Pillay D, Garnett G, et al. Effect on transmission of HIV-1 resistance of timing of implementation of viral load monitoring to determine switches from first to second-line antiretroviral regimens in resource-limited settings. *AIDS Lond Engl*. 2011;25(6):843-850. doi:10.1097/QAD.0b013e328344037a

14. Phillips AN, Venter F, Havlir D, et al. Risks and benefits of dolutegravir-based antiretroviral drug regimens in sub-Saharan Africa: a modelling study. *Lancet HIV*. 2018;6(2):e116-e127. doi:10.1016/S2352-3018(18)30317-5

15. Phillips AN, Bansi-Matharu L, Shahmanesh M, et al. Potential cost-effectiveness of community availability of tenofovir, lamivudine, and dolutegravir for HIV prevention and treatment in east, central, southern, and west Africa: a modelling analysis. *Lancet Glob Health*. 2023;11(10):e1648-e1657. doi:10.1016/S2214-109X(23)00383-2
