# Supplementary material for: Cost-effectiveness of leveraging existing HIV primary health systems and community health workers for hypertension screening and treatment in Africa: An individual-based modeling study
Source: PLoS Med. 2025 Jan 24;22(1):e1004531. doi: 10.1371/journal.pmed.1004531 (PMC11805449; doi:10.1371/journal.pmed.1004531)
Supplement: Attachment — Submitted filename: response to reviewers.docx [file pmed.1004531.s006.docx]

GENERAL EDITORIAL REQEUSTS
* At this stage, we ask that you include a short, non-technical Author Summary of your research to make findings accessible to a wide audience that includes both scientists and non-scientists. The Author Summary should immediately follow the Abstract in your revised manuscript. This text is subject to editorial change and should be distinct from the scientific abstract. Ideally each sub-heading should contain 2-3 single sentence, concise bullet points containing the most salient points from your study. In the final bullet point of ‘What Do These Findings Mean?’ Please include the main limitations of the study in non-technical language.

Please see our author guidelines for more information: [https://urldefense.com/v3/__https://journals.plos.org/plosmedicine/s/revising-your-manuscript*loc-author-summary__;Iw!!LQC6Cpwp!oHpUu-7CNV1XaWfgwP5l9HKvLTBZPXqJjeAZoYRbIKpvAcNL3sHrV4aVGzGGicbzcASrMMqYM9OX0oynFXRb$](https://urldefense.com/v3/__https:/journals.plos.org/plosmedicine/s/revising-your-manuscript*loc-author-summary__;Iw!!LQC6Cpwp!oHpUu-7CNV1XaWfgwP5l9HKvLTBZPXqJjeAZoYRbIKpvAcNL3sHrV4aVGzGGicbzcASrMMqYM9OX0oynFXRb$) .

*We have added the Author Summary.*

* Please confirm that your title complies with to PLOS Medicine's style. Your title must be nondeclarative and not a question. It should begin with main concept if possible. "Effect of" should be used only if causality can be inferred, i.e., for an RCT. Please place the study design ("A randomized controlled trial," "A retrospective study," "A modelling study," etc.) in the subtitle (ie, after a colon).

*We confirm that our title complies with PLOS Medicine’s style*

* Please confirm that your abstract complies with our requirements, including providing all the information relevant to this study type [https://urldefense.com/v3/__https://journals.plos.org/plosmedicine/s/submission-guidelines*loc-abstract__;Iw!!LQC6Cpwp!oHpUu-7CNV1XaWfgwP5l9HKvLTBZPXqJjeAZoYRbIKpvAcNL3sHrV4aVGzGGicbzcASrMMqYM9OX0vw_wrbB$](https://urldefense.com/v3/__https:/journals.plos.org/plosmedicine/s/submission-guidelines*loc-abstract__;Iw!!LQC6Cpwp!oHpUu-7CNV1XaWfgwP5l9HKvLTBZPXqJjeAZoYRbIKpvAcNL3sHrV4aVGzGGicbzcASrMMqYM9OX0vw_wrbB$)   

*We confirm that the abstract complies with PLOS Medicine’s requirements*

* Please ensure that the Introduction ends with a clear description of the study question or hypothesis.

*We confirm that the introduction ends with a description of the key study question.*

* Please ensure that all abbreviations are defined at first use throughout the text.

*We have revised abbreviations to ensure all are defined at first use.*

GENERAL
* Please provide the author contributions in full according to the format outlined for PLOS Medicine [https://urldefense.com/v3/__https://journals.plos.org/plosmedicine/s/authorship*loc-author-contributions__;Iw!!LQC6Cpwp!oHpUu-7CNV1XaWfgwP5l9HKvLTBZPXqJjeAZoYRbIKpvAcNL3sHrV4aVGzGGicbzcASrMMqYM9OX0jyAE86c$](https://urldefense.com/v3/__https:/journals.plos.org/plosmedicine/s/authorship*loc-author-contributions__;Iw!!LQC6Cpwp!oHpUu-7CNV1XaWfgwP5l9HKvLTBZPXqJjeAZoYRbIKpvAcNL3sHrV4aVGzGGicbzcASrMMqYM9OX0jyAE86c$)

*We have checked the author contributions entered into the Editorial Manager system and confirm that they are correct. We have removed the free text section of contributions in the manuscript text to avoid duplication and confusion.*

* Please adjust all references to the Appendix to be to the Supporting Information, and format accordingly [https://urldefense.com/v3/__https://journals.plos.org/plosmedicine/s/supporting-information__;!!LQC6Cpwp!oHpUu-7CNV1XaWfgwP5l9HKvLTBZPXqJjeAZoYRbIKpvAcNL3sHrV4aVGzGGicbzcASrMMqYM9OX0hL04-__$](https://urldefense.com/v3/__https:/journals.plos.org/plosmedicine/s/supporting-information__;!!LQC6Cpwp!oHpUu-7CNV1XaWfgwP5l9HKvLTBZPXqJjeAZoYRbIKpvAcNL3sHrV4aVGzGGicbzcASrMMqYM9OX0hL04-__$)  

*We have modified references to the Appendix to meet PLOS Medicine’s formatting requirements*

FUNDING STATEMENT
* The funding statement should include: specific grant numbers, initials of authors who received each award, URLs to sponsors’ websites. Also, please state whether any sponsors or funders (other than the named authors) played any role in study design, data collection and analysis, the decision to publish, or preparation of the manuscript. If they had no role in the research, include this sentence: “The funders had no role in study design, data collection and analysis, decision to publish, or preparation of the manuscript.”

*We have confirmed our funding statement.*

COMPETING INTERESTS STATEMENT
* All authors must declare their relevant competing interests per the PLOS policy, which can be seen here: [https://urldefense.com/v3/__https://journals.plos.org/plosmedicine/s/competing-interests__;!!LQC6Cpwp!oHpUu-7CNV1XaWfgwP5l9HKvLTBZPXqJjeAZoYRbIKpvAcNL3sHrV4aVGzGGicbzcASrMMqYM9OX0vH-dBet$](https://urldefense.com/v3/__https:/journals.plos.org/plosmedicine/s/competing-interests__;!!LQC6Cpwp!oHpUu-7CNV1XaWfgwP5l9HKvLTBZPXqJjeAZoYRbIKpvAcNL3sHrV4aVGzGGicbzcASrMMqYM9OX0vH-dBet$)  For authors with ties to industry, please indicate whether any of the interests has a financial stake in the results of the current study.

*All authors certify that they have no competing interests.*

DATA AVAILABILITY
* Thank you for agreeing to make your data available. At this time, please provide the link to the data repository and accession numbers required for access.

*We have submitted our code and dataset to be available for public access through Dryad, which will be publically available as soon as approved by the Dryad platform.*

*Forthcoming on Dryad.* [*https://doi.org/10.5061/dryad.5x69p8d9b*](https://doi.org/10.5061/dryad.5x69p8d9b)

FIGURES
* Please show graph axes beginning at zero. If this is not possible, please show a break in the axis.

*We have double checked all figures and confirm that axes begin at zero.*

ECONOMIC ANALYSES INCLUDING COST-EFFECTIVENESS ANALYSES
* Please report your economic analysis according to the appropriate study design provided at [https://urldefense.com/v3/__http://www.equator-network.org/?post_type=eq_guidelines&eq_guidelines_study_design=economic-evaluations&eq_guidelines_clinical_specialty=0&eq_guidelines_report_section=0&s=__;!!LQC6Cpwp!oHpUu-7CNV1XaWfgwP5l9HKvLTBZPXqJjeAZoYRbIKpvAcNL3sHrV4aVGzGGicbzcASrMMqYM9OX0hErMubw$](https://urldefense.com/v3/__http:/www.equator-network.org/?post_type=eq_guidelines&eq_guidelines_study_design=economic-evaluations&eq_guidelines_clinical_specialty=0&eq_guidelines_report_section=0&s=__;!!LQC6Cpwp!oHpUu-7CNV1XaWfgwP5l9HKvLTBZPXqJjeAZoYRbIKpvAcNL3sHrV4aVGzGGicbzcASrMMqYM9OX0hErMubw$)  and provide the relevant completed checklist. In the checklist please include sufficient text excerpted from the manuscript to explain how you accomplished all applicable items.

*We confirm that we report according to the appropriate reporting guidelines and have included the CHEERS checklist as a supplemental file.*

Comments from Reviewers:

Reviewer #1: The effort the author has put into addressing the concerns and comments raised in the previous review is appreciated.

The revised manuscript has resolved the issues previously highlighted. The author's responses to the comments were clear and comprehensive, and the additional information provided has strengthened the study's methodology and conclusions. 

Overall, the manuscript is now well-structured and provides valuable insights into the topic. I have no additional substantive concerns. 

Good luck!


Reviewer #2: Thank you to the authors for integrating my comments into the paper. I'm happy to approve for publication. 


Reviewer #3: The authors have thoroughly responded to all of my comments and queries, and have done a commendable job making further improvements to their their manuscript.


Reviewer #4: Thank you for addressing the comments in my initial review.

*We thank all the reviewers for their time and helpful comments to improve the quality of our manuscript.*
